# Potential living kidney donors’ positive experiences of an information letter from healthcare: a descriptive qualitative study

**DOI:** 10.1186/s12882-022-02959-5

**Published:** 2022-10-14

**Authors:** Eva Lagging, Kjerstin Larsson, Jonas Wadström, Linda Gyllström Krekula, Annika Tibell

**Affiliations:** 1 Center for Health Care Ethics, LIME, Karolinska Institutet, 171 77 Stockholm, Sweden; 2 Perioperative Medicine and Intensive Care, Regional Donation Center Stockholm-Gotland, Karolinska University Hospital, 171 76 Solna, Stockholm, Sweden; 3 Department of Public Health and Caring Sciences, Health equity and working life, Uppsala University, 752 37 Uppsala, Sweden; 4 Department of Transplantation Surgery, Karolinska University Hospital, 141 86 Huddinge, Stockholm, Sweden; 5 Department of Research, Education and Innovation, Karolinska University Hospital, 171 76 Stockholm, Sweden

**Keywords:** Qualitative, Living kidney donor, Transplantation, Healthcare, Information, Recruitment phase

## Abstract

**Background:**

Patients who need a live donor kidney transplant (LDKT) must often ask potential donors (PLDs) themselves. This is a difficult task and healthcare could unburden them by making this first contact, ensuring also that PLDs receive correct information. We investigated how PLDs experience receiving a letter from healthcare about LDKT, live kidney donation, and inviting them to meet with professionals to get more information.

**Methods:**

The letter (LD-letter) was sent to a cohort of 46 individuals, from which a purposeful sample of 15 were interviewed using a semi-structured guide covering their experience of the letter, views on being approached by healthcare, and opinions on style and content. Interviews were analyzed using conventional inductive analysis.

**Results:**

We identified three categories of experiences: Category (1) Reflections on receiving the letter, contains three subcategories relating to how the letter did not induce pressure to donate, did not affect the PLD’s relationship with the patient with kidney disease, and made the letter-receiver feel important in the transplant process; Category (2) The letter creates clarification and trust, also contains three subcategories, relating to how it clarified the voluntariness of donation and neutrality of healthcare providers with respect to the PLD’s decision, elucidated the patient with kidney disease’s current stage of disease (where transplantation was approaching), and unburdened patients from the responsibility of contacting PLDs on their own; Category (3) Opinions and suggestions about the letter and further communication, with four subcategories, relating to preference of a letter as the first step for communication about LDKT, suggestions on style and content, views on following up the letter, and how open meetings about LDKT were an important information source. Furthermore, 80% of the interviewees found the letter’s information comprehensive, 67% found it easy to read and respectful, and 86% rated it as good or very good.

**Conclusion:**

Potential donors prefer and recommend a letter as the first step for communication regarding LD. The LD-letter unburdens patients from the task of asking PLDs and stresses the voluntariness of donation, does not leave PLDs feeling coerced or lead to negative effects in their relationship with the patient.

**Supplementary Information:**

The online version contains supplementary material available at 10.1186/s12882-022-02959-5.

## Introduction

Kidney disease is a growing health problem around the globe and the number of patients with end-stage renal disease (ESRD) is increasing [1]. Kidney transplantation is generally the preferred treatment as it increases life expectancy and improves the quality of life as compared to dialysis [2]. Transplantation can be done with kidneys from deceased donors (DDs) or living donors (LDs). Living donor kidney transplantation (LDKT) has the advantage of enabling transplantation before dialysis is needed as well as having superior results to DD transplantation [3, 4].

While the number of DDs has increased in recent years in many countries, including Sweden, the number of LDs has not shown a similar trend and the need for kidneys for transplantation continues to far exceed availability [5]. The median waiting time in Sweden for a DD kidney is currently 11 months, however this does not include highly sensitized patients [5].

When it comes to approaching potential living kidney donors, the most common practice is for patients with kidney disease to contact potential donors (PLDs) themselves [6]. Approaching potential donors and explaining LDKT is a complex task that requires more than one ability. Having sufficient knowledge about LDKT and having the ability to provide information to others, however, are key [7]. Many patients do not want to ask potential donors themselves, but instead would like LDs to come forward spontaneously [8]. Some of the reasons for this given by patients are that the potential donor may feel pressured or may be harmed by donation, and that the patient finds it difficult to approach family and friends about needing a donated kidney [8].

There is a growing awareness that patient with kidney diseases need professional support with the task of finding a LD [9]. To date, several strategies have been developed to help patient with kidney diseases with this difficult task and to increase access to living kidney donation (LKD), such as home-based education (HBE), smart phone applications, and talk about live kidney donation (TALK) [10]. These interventions target patient with kidney diseases and their social networks to increase their knowledge about LKD.

A decision to become a living kidney donor must be made of one’s own free will and without coercion. It is therefore important to know how those who receive the LD-letter perceive being informed by healthcare about living kidney donation via a letter. To our knowledge, this method of informing potential kidney donors has not previously been investigated or described. Thus, the aim of this study was to investigate how people close to a person with kidney disease experience receiving the LD-letter.

## Methods

### Study design

As interviews are useful in investigating people’s experiences and feelings, we conducted an interview study with a qualitative design using a descriptive approach [11]. The reporting was carried out according to the Consolidated Criteria for Reporting Qualitative research (COREQ), a well-established checklist for qualitative studies [12].

### Setting

The LD-letter was developed at meetings where staff from the nephrology and transplantation departments as well as patients and living kidney donors were represented. A professional language examiner then reviewed the text to ensure it was neutral and easy to read and understand.

The Department of Nephrology at Danderyd Hospital, a university hospital in Stockholm, Sweden, chose to send the letter (LD-letter) to relatives and close contacts to individuals with kidney disease to inform them about kidney transplantation and the possibility of live kidney donation. The LD-letter contained information about how the patient needs active replacement therapy and that transplantation is the preferred treatment. The letter also informed about how a transplanted kidney could come from a deceased or live donor, and that LDKT is advantageous. Finally, the letter contained information about how healthcare professionals were a neutral party and respect everyone’s decision regarding donation and that the PLD’s decision should be based entirely on free will. The letter-receiver was offered more information about living kidney donation (LKD) from healthcare professionals if desired.

In Sweden, evaluation of potential LDs is conducted by nephrology departments. The nephrologist assessing a living donor does not usually have a therapeutic relationship with the patient with kidney disease. The possibility of sending a LD-letter to PLDs was raised with the patient when his or her glomerular filtration rate (GFR) was around 15–20 mL/min/1.73m^2^. This usually occurred at the same time as future and possible treatment options were discussed, i.e., when discussing transplantation as the best option. Approval to send the letter, and to whom, was obtained from the patient with kidney disease by the patient’s doctor. The sender of the LD-letter was the Department of Nephrology, and a brochure about living kidney donation accompanied the letter. Sending the letter at this time-point meant that the patient’s need of dialysis or transplantation had not yet become urgent. Hence, there was still time for the receiver of the letter to become informed and consider the idea of becoming a living kidney donor.

### Participants

The total population consisted of 49 individuals who had received the LD-letter from the Department of Nephrology at Danderyds Hospital, Sweden (Table 1). A purposeful sampling was then used to ensure variation in participant gender, relation to patient with kidney disease, and the year the LD-letter was received. Interviewees were contacted a few at a time according to the sampling strategy and received written information about the background and purpose of the study. This initial contact was followed by a telephone call a week later, during which a time and place for those interested in being interviewed was decided. Once saturation had been reached, after 31 potential interviewees had been contacted and 15 interviews were completed, the interviewing stopped. The remaining 18 individuals who had received the LD-letter from healthcare were therefore not contacted for interviews.

Fifteen of the people contacted declined to participate in the study, 10 women and 5 men. These people’s relationship to the patient varied, as did the year they received the LD-letter (Table 1). Several different reasons were given for not participating, but the overall theme was that the issue was not relevant at this time. Reasons given included the letter-receiver’s own- or family health issues, lack of time, changed family situation, and/or the patient with kidney disease was deceased or transplanted. One person who initially agreed to participate was not interviewed due to changed life circumstances. The final sample thus consisted of 15 participants, 9 women and 6 men, with varying relationship to the patient and year the LD-letter was received (Table 1).

**Table 1:** Participant characteristics

	Interviewed (n = 15)	Declined participation (n = 15)	Total population who received LD-letter (n = 49)
**Gender**	9 women/6 men	10 women/5 men	30 women/19 men
**Age** ^**(a)**^			
- 41–50	2		
- 51–60	5		
- 61–70	6		
- > 70	2		
**Relationship to patient with kidney disease**			
- Parent	5	2	9
- Sibling	6	4	20
- Partner	3	4	10
- Child	0	3	5
- Friend	0	1	2
- Other	1	1	3
**Year received letter**			
- 2013	0	2	3
- 2014	3	3	13
- 2015	5	4	17
- 2016	3	3	6
- 2017	0	2	3
- 2018	3	2	6
- 2019	1	0	1

(^a^) Information about age was collected via a questionnaire that the participants answered during the interview. Thus, information about age is not available for non-participants.

### Data collection

To guarantee the same topics were covered with all participants, data were collected using a semi-structured interview guide consisting of open-ended questions. As all the interviewees were native Swedish speakers, the interviews were conducted in Swedish. The interview guide included questions about the participant’s first impression of the letter, opinions on the style of the letter (i.e., its tone, readability, content, and layout), views on being approached by healthcare, contact with the recipient, and thoughts about LKD. The participants also answered a short questionnaire that included individual participant characteristics and overall ratings of the LD-letter (see supplementary material). The interview guide was pilot-tested on two previous kidney donors and two potential living kidney donors, all of whom had received the LD-letter prior to conducting the pilot test. Minor changes were made in the interview guide after this testing.

All interviews were face-to-face and conducted by author EL. To facilitate the PLDs’ participation in the study, they were given the choice of being interviewed at their home or another agreed-upon location. Six interviews took place at the participant’s home, six in a hospital meeting room, two at the interviewer’s office, and one at the participant’s office. Only the participant and first author were present during the interviews. The interviews were audio-recorded, with a mean interview time of 60 min (min. 37 – max. 91). All interviews were transcribed verbatim, resulting in a total of 252 pages.

### Analysis

The qualitative data was analyzed using conventional content analysis, which is suitable when the aim of the study is to describe a phenomenon where existing theory or research literature on the phenomenon is limited [13]. The analysis was carried out on the untranslated transcriptions, which were in Swedish.

All interviews were read and reread to capture the essence of the text. Meaning units, i.e., text that captured key thoughts or concepts of the study aim, were identified, and labeled with codes that described their content. The codes were then compared for similarities and differences and grouped into a structure of categories and subcategories. The category and subcategory groupings were discussed by EL and KL until a shared understanding of the results was reached. Table 2 illustrates an example of this process. During the analysis process, the authors involved constantly moved back and forth between the parts – such as the codes – to the entire text to ensure coherence. This preliminary classification structure was then discussed among the research group until agreement about the relevance of the categorization was reached. In a final step, citations were chosen to illustrate the content of the categories. No computer software was used for analysis and data storage, nor was member checking performed. The quantitative data was processed by calculating percentages.

**Table 2:** Example of qualitative analysis from meaning unit, code, subcategory to category

Meaning unit	Code	Subcategory	Category
…precisely, that it addresses this by stating that a person’s decision should be made of their own free will and no one should be coerced into wanting to donate or, more or less, have a bad conscience if they don’t. That is very important.	My decision	Clarifies the decision is that of the letter-receiver	The LD-letter creates clarification and trust

### Research team

The authors pre-understanding of the phenomena differed. The lead author EL is a PhD student with experience of interviewing for patient associations. Authors KL and LGK are medical social workers and qualitative researchers, in chronic diseases and deceased organ donations, respectively. Authors JW and AT are transplant surgeons, the former with a focus on LDKT and the latter on organ donation. Authors KL, LGK, JW and AT are all PhDs.

#### Contact with participants

Only the main author had contact with the participants and this contact was in connection with the study.

### Ethical considerations

The interview questions may be perceived by the participants as personal and could thus constitute an infringement of their integrity.

All participants were informed, both in written form and orally, that participation was voluntary, and that the participant had the right to withdraw at any time without explanation. Participants were also informed that the study material would be pseudonymized and remain strictly confidential throughout the study process. Thus, a particular response cannot be linked to a specific participant. Prior to the interview, written consent was obtained from the participants. Should they need further support, all participants were also offered contact with a social worker. None desired this.

The study was approved by the Ethics Review Committee in Stockholm (ref. no.: 2016/2450-31/1). This study contains NO organs/tissues procured from prisoners.

## Results

The following section presents the results from the qualitative analysis and an overall rating of the LD-letter.

The analysis of the transcripts resulted in three categories and a number of subcategories: Category (1) Feelings evoked bythe LD-letter, with three subcategories; Category (2) The LD-letter creates clarification and trust, with three subcategories; and Category (3) Opinions and suggestions about the LD-letter, with four subcategories. An overview of the categories and subcategories is presented in Table 3.

**Table 3:** Relationship between categories and subcategories

Category	Subcategories
Feelings evoked by the LD-letter	The LD-letter does not induce pressure to donate. The LD-letter does not affect the relationship between the potential donor and the patient. The LD-letter makes the receiver feel important in the transplant process.
The LD-letter creates clarification and trust	The LD-letter clarifies that the decision to volunteer as donor is the letter-receiver’s decision.
	The LD-letter clarifies the patient’s phase in the transplant process.
	The LD-letter unburdens the patient from approaching and informing potential donors.
Opinions and suggestions about the LD-letter and further communication.	A letter is preferred as the first step for communication regarding LKDT. Opinions and suggestions regarding style and content of the LD-letter.
	Opinions and suggestions regarding follow-up of the LD-letter.
	Need for meetings about LKDT.

### Category 1. Feelings evoked by the LD-letter

#### The LD-letter does not induce pressure to donate

The study participants felt that the LD-letter was undramatic and that it did not induce feelings of pressure to donate or leave them with guilt or a bad conscience if they chose not to donate a kidney. This view was found in the interviews regardless of the participant’s willingness to donate or not.

*I don’t think that the letter, through its wording or anything, put me in any, that I felt pressure or anything, is free from requirements. It’s only info about what it entails if you have thought about this and, are interested in finding out more. (10M)*

#### The LD-letter does not affect the relationship between the potential donor and the patient

The participants were asked if the LD-letter had affected their relationship with the patient with kidney disease. All participants expressed that the relationship with the patient with kidney disease was not changed in any way by the LD-letter.

*We have always had a good relationship and we still do* (after receiving the LD-letter). *(1K)*

#### The LD-letter makes the receiver feel like an important person in the transplant process

The fact that the LD-letter was addressed to them personally, by name, and sent to their home address, made them feel chosen and an important part of the transplant process, thus making them feel respected. The letter also made them feel cared for and that they were getting attention from healthcare.

*At the same time, the letter fulfills the function that someone cares about me too. So now I´m getting some attention when I get this. Someone sees me too. (12 K)*

The participants found it important that the letter explained that the patient with kidney disease had chosen them as the receiver of the letter.

*We have received, the patient’s name is there, that is also an important part mentioning you as a possible. Because then you feel a little more hand-picked, I also think, that he has sort of mentioned me in this. (1K)*

### Category 2: The LD-letter creates clarification and trust

#### The LD-letter clarifies that the decision to volunteer as donor is the letter-receiver’s decision

The sentences in the LD-letter that were consistently emphasized by the participants as being most important were those that highlighted that the decision to become a LD should be voluntary, based on free will, and entirely their own, and that healthcare was neutral in relation to their decision (see appended LD-letter). These sentences also provided a sense of security and trust. Receiving a LD-letter specifically addressed to the participant, containing written information and signed by several healthcare experts in the field, helped to reinforce their perception that the decision to donate was their own.

*It addresses the fact that it is based on free will and that people should not be coerced into wanting to or, more or less, be left with a bad conscience if they don’t donate. That is very important. (6M)*

#### The LD-letter clarifies the patient’s phase in the transplant process

Receiving the LD-letter made it clear to the participants that the patient with kidney disease was approaching a stage when they would need dialysis or a kidney transplant.

(The patient) *is so sick or, this letter comes when the time for dialysis or donation is starting to approach. (8K)*

The participants also stated that, through the LD-letter, they became aware that different activities related to the new phase the patient was entering, such as medical evaluation and dialysis, could also have an impact on them due to limitations to their freedom and loss of energy that dialysis often causes in a patient with kidney disease.

#### The LD-letter unburdens the patient from approaching and informing potential donors

Participants appreciated that healthcare professionals were taking an active part in contacting potential donors. They considered healthcare to be a neutral party since they acted from a professional standpoint, which the participants mentioned as positive and important. They pointed out that it would be too much to expect the patient with kidney diseases themselves to inform about LD, and they felt that the patient with kidney diseases should not be burdened with that role.

*It’s really great, I think, that healthcare takes a more active part in this contact because it’s really awful that the patient with kidney disease should do it. It’s great because healthcare is a neutral party that can act from a professional standpoint. (2K)*

### Category 3: Opinions and suggestions about the LD-letter and further communication

#### A letter is preferred as the first step for communication regarding LKDT

The participants considered a letter to be the optimal way to convey information about living kidney donation and preferred a posted letter in paper format rather than other means of communication.

*I think it feels reassuring when you get a written document, you feel that things are in order, which is important in a process like this. (2K)*

The participants stated that the most important advantage of using a letter as the method of communication was that it gave them the opportunity to read it, undisturbed, in their own home and at their own pace. They thought, in addition, that it was valuable to be able to refer back to the letter and reread it whenever they felt the need.

*Where you can go back and look at it. The best is in paper form, I think (13K)*

*I think a letter is good. You can’t take up things like this on the phone, it has to come so that you can read it yourself – once or twice, or three or ten times if you need to. (1K)*

They also appreciated that the letter informed about the possibility to discuss their thoughts and concerns about LD with a healthcare professional.

#### Opinions and suggestions regarding style and content of the LD-letter

The participants stated that the LD-letter was written in cordial and respectful tone. They also found it easy to read and objective. Furthermore, the informants found the LD-letter to be informative, factual, and neutrally written, using everyday language, without medical terms, and that it referred to further information regarding LD. They felt that the LD-letter content was adapted to the receiver, which was appreciated by the participants.

*It’s easy to read, it’s quite a lot. It’s neutral but I would also call it respectful, and I think that’s important. (5M)*

The participants also had some suggestions for improvements of the LD-letter, such as adding a current date and signature of at least one of the caregivers named in the letter, and that it provided a reference to a trustworthy website where they could find out more.

Others suggested improvements pertaining to different aspects of LKD, such as information about there being two different healthcare teams: one for the potential LD, focused on them and their well-being; and another for the patient with kidney disease. Additional possible improvements noted were that the LD-letter should inform them that a thorough health evaluation of the LD would be carried out to ensure that the donor was healthy enough to donate, that it is a routine operation, and about potential risks for the LD. Also, participants expressed a lack of information about the fact that, with a LD, a kidney transplant can be done so that dialysis is not needed.

*But I think it should be clearer that there is a special* (healthcare) *team that is independent, and they are on your side, so to speak. The others are on the patient’s side. “We’re on your side; we’ll do everything we can to make you feel safe.” So that [potential donors] dare to discuss things with someone who understands the problem of being a donor, and that’s the whole purpose. (3M)*

#### Opinions and suggestions regarding follow-up of the LD-letter

The participants were asked if they thought the LD-letter should be followed up and, if so, who should be responsible for making contact, they themselves or healthcare providers. Some participants felt reluctant to call the healthcare people themselves and felt it was healthcare that should be responsible for following up and making contact. They wanted to be asked whether they had received the letter and whether they had any questions regarding it and its content. However, they did not want their possible willingness to donate a kidney to be discussed at the follow-up call.

*It´s very good to follow up afterwards, maybe a call afterwards, after you’ve received a letter. (9K)*

Other participants stated that they themselves should be responsible for following up the letter and wanted to do so when they were ready with their thoughts and questions. They felt that if healthcare provided the follow up, it could be perceived as pressure or intrusive.

*No, I probably think it’s better that, in this case, I contact them when I’m ready with my questions and thoughts. It’s better because I think there might otherwise be a pressure effect if the healthcare staff calls. (2K)*

There were also participants who could not decide whether it should be they themselves or the healthcare provider that should follow up the letter. They mentioned the same pros and cons as stated above.

#### Need for meetings about LKDT

Meetings about living kidney donation with the participation of both healthcare providers and people who have undergone LD as donors, and preferably also recipients, was seen as an important source of information. They mentioned that people with personal experience of being a LD can convey dimensions and perspectives other than the medical aspects that healthcare staff provide. These types of meetings are already offered today, and the participants pointed out how important they had been for them as a source of information. Thus, they wanted the LD-letter to include information on dates, times, and places for upcoming LD meetings.

*So, at hospitals they had theme nights where we were invited and where also people who had received a kidney and their living donors talked about their experiences. It was very interesting. (4K)*

### Overall rating of the LD-letter

Most of the participants gave the LD-letter a high rating. With regard to information, 80% found it comprehensive. When it came to the text and tone of the letter, 67% found it easy to read and respectful. No one thought the LD-letter was difficult to read or insensitive. Regarding the overall rating of the LD-letter, 86% found it good or very good, while one participant found it neither good nor bad, and one fairly bad. No one rated it as very bad. (See Table 4.)

**Table 4:** **Overall rating of the letter**

	n=
**The information was**:	
- Too comprehensive	1
- Appropriately comprehensive	12
- Too brief	2
**The text was**:	
- Easy to understand	10
- Okay	5
- Difficult to understand	0
**The tone was**:	
- Respectful	10
- Neutral	5
- Insensitive	0
**Overall rating of the letter**:	
- Very good	8
- Fairly good	5
- Neither good nor bad	1
- Fairly bad	1
- Very bad	0

## Discussion

To our knowledge, there are no previous studies about this method of informing potential LDs. The intention of the LD-letter is to inform about kidney transplantation and living kidney donation, and to facilitate a first contact between a patient with kidney disease and potential donors. The letter does not constitute or include a comprehensive guide to the process. Nor does it attempt to weigh the risks and benefits of kidney donation in order to facilitate the decision-making process.

In the Swedish kidney donation context, arguments from healthcare providers averse to sending the LD-letter included that such a letter would put pressure on the receivers to register as potential LDs and that it would negatively affect the relationship between the patient with kidney disease and receiver of the LD-letter. The mentioned serious fears are the reasons why we wanted to study how the letter was experienced by PLDs. These concerns were not confirmed by our study, however. On the contrary, our findings show that the LD-letter was not perceived to entail any pressure or invasion of privacy, or to affect the relationship between the patient with kidney disease and letter-receiver, which is an important message from potential donors. Receiving a personally addressed letter to their home made the participants feel chosen, respected and as important in the transplant process. It was also important that the letter explain that the patient with kidney disease him/herself had chosen them as the receiver of the letter.

The LD-letter creates clarification and trust in that the decision to donate is the potential donor’s to make and that the decision must be voluntary. Similar results were found by Brown et al., where kidney donors stated that their decision to donate was personal and that they must feel comfortable with their decision because it is the donor who has to live with it [14]. Voluntarism is, in addition, an important aspect of the concept of informed consent as well as of international guidelines and policies on evaluation and care of LDs [15–17].

The medical team is thus responsible for ensuring that a potential donor’s decision to donate is voluntarily and that they are free to withdraw that decision at any time [15–17]. Raising the issue of LD via the LD-letter enables the healthcare provider to take an active role at an early stage in the LKD process. The earlier healthcare can provide accurate information, the more likely it is that potential LDs will be able to use that information in making their decision and prevent a decision based on misconceptions regarding LKDT. According to Ummel et al., a LD’s decision-making process often begins before their donor evaluation commences [18]. This means that the decision process may begin before potential donors meet healthcare providers working with living kidney donation who can provide them with relevant and accurate information [18]. Agerskov et al. found that good communication between healthcare and the donor increased predictability, confidence, motivation, and commitment of the donor, which in turn promoted optimal post-donation outcomes [19].

Our participants considered it positive that healthcare providers are a neutral part acting from a professional standpoint. In our study, this was mentioned from several aspects. Information about healthcare providers being neutral with respect to the PLD’s donation decision, regardless of whether that decision was affirmative or negative, was appreciated. Healthcare’s neutrality also facilitated the opportunity (mentioned in the LD-letter) for potential LDs to ask questions and discuss LKDT issues with healthcare professionals. The importance of enabling PLDs to discuss LDK with healthcare professionals is supported by A recent study by Schick-Makaroff et al. supports our finding. [20].

Healthcare’s neutrality and knowledge about LKD are also important as our study participants note this as a reason why healthcare providers should have the responsibility to contact and raise the issue of LD with potential donors. This in turn relieves the patients from handling this on their own. At present, the most common practice for approaching potential LDs is for patients with kidney disease to make these contacts themselves. This was also found by Mazaris et al., who describe how healthcare providers, to a much higher degree than patient with kidney diseases or LDs, feel that the initial approach should be made by the patients [6]. However, more in line with our results, a previous study found that patient with kidney diseases seeking a LD required guidance and support, that they found it difficult to approach their social network regarding their need for a LD, plus that they did not know where to turn for help [21].

Even patient with kidney diseases who received coaching to discuss their need of a kidney found it difficult to approach others about live donation [9]. Thus, educating patient with kidney diseases about LKDT gives them more knowledge, but does not necessarily lead to more completed LKDTs [22]. Furthermore, patient with kidney diseases vary in their ability to approach potential LDs and inform them about the medical aspects [23].

When the participants were asked about follow-up of the LD-letter, some wanted the healthcare providers to be responsible for this, while others stated that they would perceive this as being pressured and would instead prefer to contact the healthcare team themselves. None of the participants wanted their potential willingness to donate a kidney to be addressed on this occasion. One way of handling the follow-up could therefore be to include a card with the LD-letter asking if the PLD would like to be contacted by healthcare. Those who want to be contacted can signal this by returning the enclosed card. Healthcare can then contact only those PLDs who have accepted this via the card, and not the others.

Most of the participants found the LD-letter to be objective, respectfully written, and easy to read as they felt it was written in everyday language with no medical terminology used. They also considered its content to be adapted to the receiver. The importance of formulating information to potential LDs at a level that facilitates their comprehension of the information has been shown in previous research [24]. Potential LDs level of health literacy may influence their ability to understand and use information about LKD [25]. Thus, keeping the information simple and using everyday language may make the LKD process more accessible to a wider range of people [26].

All participants preferred to initially receive the LD-letter and then receive other types of information, such as through LKDT meetings. Those who had experience of LKDT meetings, where both healthcare providers and individuals who had previously donated a kidney or received one were present, expressed great appreciation of these meetings. The meetings were considered an important source of information and the opportunity to attend anonymously was appreciated. Another study found LKDT meetings (which they called “Saturday seminars”) useful in informing both patient with kidney diseases and members of their social network about LKDT [27].

The importance of potential LDs having access to information from individuals with personal experience of the LKD process has also been shown in other studies [20, 21, 28]. These individuals convey dimensions and perspectives that add to the factual and medical aspects provided by the healthcare staff. They have faced the same challenges and decisions and can therefore address fears, emotional issues, offer practical advice, and share experiential knowledge related to considering LD, in a way that only someone with personal experience of donation can do [19–21, 29]. Another study showed that access to a mentor, pre-donation, provided donors with greater confidence in their own decision to donate [28]. Other information methods targeted at both patient with kidney diseases and their social network are home-based education interventions (HBE) and Talk About Live Kidney Donation social worker interventions (TALK-SWI) [30–32]. In HBE, information is given in a homelike environment by healthcare personnel knowledgeable in the field. The TALK-SWI method consists of a first meeting focused on helping patients identify barriers to their considering LDKT, followed by a second meeting that also includes the patients’ social network, to help identify barriers to pursuing LDKT. One difference between these other communication methods and the LD-letter is that, while the other methods to a large extent make the patient responsible for sending out invitations to such meetings to their social network, in the case of the LD-letter, it is the healthcare providers who handle the invitation. In addition, the LD-letter enables people residing across a larger geographical area to receive information. And because it does not require large resources, even smaller clinics can use the LD-letter method.

Having accurate information and interactive applications regarding LDKT available on the internet provides potential LDs, especially those who are hesitant, easy access to it before meeting with healthcare professionals. Reliable online resources may also help to address misconceptions and fears the donor may have and enable them to consider and deliberate donating prior to disclosing their interest to others [33, 34]. A lack of knowledge has been identified as a factor that may prevent or discourage potential LDs from becoming an organ donor [21, 35]. Instead of face-to-face meetings, Waterman and colleagues have described a web-based project to present personal stories from LDs and kidney recipients that could be used to inform interested parties – everyone from PLDs to the general public [36].

Several measures were taken to strengthen the credibility of the study findings. As noted, a purposeful sampling was performed among persons who had received the LD-letter from healthcare, in order to attain variation in participant gender, relation to patient with kidney disease, and the year the LD-letter was received. An interview guide with open-ended questions was moreover used and, prior to the study, a pilot test of the guide conducted on previous and potential living kidney donors. Finally, the first steps of the analysis were performed separately by the first and second author, who had different pre-understandings of transplantation and organ donation, whereupon further discussion with the entire research group followed, until the assigned codes and categories were consistent. The first, third, fourth and fifth authors’ pre-understanding of organ donation contributed to both broaden and deepen the analysis of the data. The second author had no prior experience of working with organ donation, thus strengthening the analysis by minimizing the impact of the other authors’ pre-understanding. The study´s dependability was enhanced by a transparent description of the steps in the research process. To achieve confirmability, authentic citations were used to illustrate the content of the categories. Transferability was promoted by using an inductive approach during analysis. The results thus represent a wide spectrum of the participants’ experience and may also be valid for others who have a similar relationship to a patient with kidney disease.

There are limitations to this study that should be considered. These include the sampling procedure, which was not successful with respect to relationship to the patient with kidney disease, in that the sample lacked relations such as children and friends of the patient, and the year the LD-letter was received, in that no participants who received the letter in 2013 and 2017 took part. The large variation in the number of letters sent per year is probably partly due to that use of the LD-letter is not a routine practice in healthcare. In addition, it was the patients with kidney disease themselves who decided to whom and to how many people the letter should be sent. In the case of 2019, the interview was conducted at the beginning of the year when this study ended.

Future research should investigate how patients with kidney disease feel about this approach to contacting potential donors. For example, what influences the patients when selecting recipients of the letter. Another area that could be studied is how the letter is perceived by individuals who are not as close to the patient and who barely know that the patient has kidney disease. Further knowledge and a better understanding of possible cultural differences are also needed. It is also important to understand how healthcare professionals selects patients they ask about sending the letter and how the patient is asked to select recipients of the letter. A randomized control study should also be done.

## Conclusion

All participants in the study felt that the letter should be used, and none felt that it induced pressure to donate or had a negative influence on their relationship with the patient with kidney disease. The letter was considered comprehensive enough, easy to read, and respectful. It also gave them a sense of security and trust by emphasizing that the decision to donate was entirely voluntary, and that healthcare providers are neutral regardless of what the potential donor decides. Furthermore, the LD-letter provides an opportunity to reach potential donors without the patient with kidney disease being responsible for making that contact themself. Certain improvements of the letter were, however, suggested by the participants. The study shows that healthcare providers can use the LD-letter to approach potential LDs, and that the letter-recipients appreciated the information contained in the letter. Future studies should also include a wider range of donors and those from different cultures as receivers of the LD-letter.

## Electronic supplementary material

Below is the link to the electronic supplementary material.

English translation of LD-letter.

The interview guide including survey questions.

## Data Availability

The data used and analyzed in this study is not publicly available because the interview transcripts contain personal and potentially identifying information. Data from participant interviews is available from the corresponding author upon reasonable request.

## Abbreviations

COREQ: Consolidated criteria for reporting qualitative research.
DD: Deceased donor.
ESRD: End-stage renal disease.
GFR: Glomerular filtration rate.
HBE: Home-based education.
LD: Living donor.
LDKT: Living donor kidney transplantation.
LKD: Living kidney donation.
PLD: Potential living donor.
TALK-SWI: Talk About Live Kidney Donation-Social worker intervention.
