# Supplementary material for: Potential living kidney donors’ positive experiences of an information letter from healthcare: a descriptive qualitative study
Source: BMC Nephrol. 2022 Oct 14;23:332. doi: 10.1186/s12882-022-02959-5 (PMC9569060; doi:10.1186/s12882-022-02959-5)
Supplement: Supplementary file 1 — English translation of LD-letter. [file 12882_2022_2959_MOESM1_ESM.docx]

**When someone close to you becomes ill – a letter about kidney donation**

**You are receiving this letter because someone close to you has kidney disease and the time when kidney transplantation may be necessary is approaching. …………… *(kidney patients name)* has given us permission to send you this letter.**

The intention of the letter is to provide information about what kidney donation entails.

We know from experience that a person in need of transplantation does not always tell the people around them about this need. Sometimes this can be partly due to the ill person not wanting a close friend or family member to feel obligated to donate a kidney.

We also know that many friends and family members would voluntarily consider becoming a donor, but no one has asked them, or they don’t know enough about what is involved with kidney donation to raise the issue themselves.

In kidney transplantation, there are two ways to get a new kidney – a donation from a person who has died or from a living donor.

When it comes to a deceased donor, the person expressed their will to donate their organs upon death before they died. A patient who is waiting for a kidney from a deceased donor is placed on a waiting list, which normally involves a waiting time of several months to a year. While they wait, the patient is treated with dialysis.

When a kidney is donated from a living donor, for example, a close friend or family member who wishes to voluntarily give the patient one of their kidneys, the transplantation can be planned in advance, which generally leads to better results.

**Living donation**

In so-called “living donation”, the living donor donates one of their kidneys and lives the rest of their life with one kidney instead of two. A decision to become a living kidney donor is based on knowledge and consideration of the process and made freely by the potential donor. The decision to donate must not be influenced by guilt or pressure from anyone or anything, and it must always be very clear to the person who wants to donate what is involved with being a donor.

In a situation like this, when the subject is so charged with emotion, it is often helpful to talk to a neutral party, to ask questions and discuss what is involved. As healthcare providers, we are entirely neutral. We have the medical knowledge about what is involved with donation and are here to provide potential donors with a neutral party to talk to. We respect every decision without question, and do not take a side for or against donation. With the help of our experience and medical knowledge, a person who is considering becoming a living donor can themself gain a sufficient basis to make a decision about what they want to do.

**Where to turn for more information**

There are several ways to find out more:

- Call the *Donation Helpline*. We have set up a special telephone line that you can call for more information. *Donation Helpline* callers who want to discuss kidney donation can do so anonymously. Telephone: 020-67 77 77
- You can contact us directly at the following telephone number:
  - Donation Nurse Louise Ax, telephone: 08-123 583 31
- There are also a number of books you can read on the subject:
  - *Morgongåvan*, by Ingela Fehrman-Ekholm. ISBN 9789163345685
  - *Den är rosa, levande: en anonym njurdonation*, by Johan Appelberg. ISBN 9185671061
- In the brochure you received with this letter you will also find a summary of information about live kidney donation. If you would like more copies of the brochure to share with others, you can order them free of charge by calling: 08-123 586 31
- You can also get more information by attending one of our information evenings. For dates and times of these meetings, please contact the Donation Nurse at the number given above.

**What can you do?**

When someone close to you becomes ill, it is common that you want help in some way. When their illness involves kidney transplantation, there are different ways you can help with donation.

- If you would like more information about kidney donation, please call or send us your name, personal identification number, and contact information. We will then set up a time for a one-on-one meeting with a doctor where you can get more information and have the opportunity to talk about things surrounding a possible decision to become a living donor. You can reach us at:
- Renal Medicine Clinic contact information:
  - Danderyd Hospital, Department of Nephrology, 182 88 Stockholm
  - Telephone: 08-123 573 30
- Another way to help is to spread knowledge about living donation to others around your close friend or family member with kidney disease.

We thank you for taking the time to read this letter. If you have thoughts or further questions, please don’t hesitate to contact us.

Stockholm, 2014

Best regards,

| ……………….. (*Name*) Head of Department Department of Nephrology Karolinska University Hospital | ……………….. (*Name*) Head of Department Department of Transplantation Surgery Karolinska University Hospital |
| --- | --- |
| ……………….. (*Name*) Head of Department Pediatric Medicine 1 Astrid Lindgren’s Children’s Hospital Karolinska University Hospital | ……………….. (*Name*)  Senior consultant Department of Transplantation Surgery Karolinska University Hospital |
| ……………….. (*Name*) Head of Department Department of Nephrology Danderyds Hospital |  |
