# Supplementary material for: Potential living kidney donors’ positive experiences of an information letter from healthcare: a descriptive qualitative study
Source: BMC Nephrol. 2022 Oct 14;23:332. doi: 10.1186/s12882-022-02959-5 (PMC9569060; doi:10.1186/s12882-022-02959-5)
Supplement: Supplementary file 2 — The interview guide including survey questions. [file 12882_2022_2959_MOESM2_ESM.docx]

**Interview guide and survey questions LD-letter**

***Questions before interview that are received in writing***

**1. Gender:**

❏ Female ❏ Male

**2. Age:**

❏ 20-30 years ❏ 31-40 years ❏ 41-50 years

❏ 51-60 years ❏ 61-70 years ❏ over 70 years

**3. Marital status:**

❏ Living together with spouse/partner ❏ Living separately from spouse/partner

❏ Single ❏ Other, what_______________

**4. What is your relationship to the recipient?**

("The recipient" in the interview = the person who needs a kidney)

I am:

❏ Spouse/partner ❏ Parent ❏ Sibling

❏ Other relative ❏ Good friend ❏ Workmate

❏ None of the above, my relationship is ___________________________

**5. Do any of the statements below apply to you today?**

❏ I have contacted the kidney clinic about possibly donating a kidney

❏ I volunteered to be a donor but was not accepted

❏ I am being evaluated as a potential living kidney donor

❏ I am approved as a living kidney donor

❏ I have donated one of my kidneys to the recipient

❏ I have not contacted the kidney clinic about possibly donating a kidney

**6. The experience of the letter**

**6a. The information was:**

❏ Too comprehensive ❏ Appropriately comprehensive ❏ Too brief

**6b. The text was:**

❏ Easy to understand ❏ Okay ❏ Difficult to understand

**6c. The tone was:**

❏ Respectful ❏ Neutral ❏ Insensitive

***Interview guide***

Tell me about your reaction when you first receiving the letter.

Describe why you consider that the information/text/tone was …………..(participants answer). (Follow-up to survey question 6a-c).

Tell me about your views on being approached by healthcare providers via a letter.

What thoughts arose in you about living kidney donation after you received the letter?

Did the letter make you act in a particular way/do something in particular – if so, describe.

Can you tell me about your relationship with the recipient and if the letter has affected it in any way.

What influenced your decision regarding donating a kidney in life? How did the letter affect your view regarding donating a kidney?

Finally, now that we have reasoned in depth about the letter - what rating do you give the letter as a whole? Describe briefly why you give that rating?

❏ Very good ❏ Fairly good ❏ Neither good nor bad

❏ Fairly bad ❏ Very bad

Do you have any other advice, views or concerns that were not covered during the interview, which you would like to express, regarding donating a kidney in life.
